# Supplementary material for: Restoring small water bodies to improve lake and river water quality in China
Source: Nat Commun. 2025 Jan 2;16:294. doi: 10.1038/s41467-024-55714-9 (PMC11697070; doi:10.1038/s41467-024-55714-9)
Supplement: Supplementary file 1 — Supplementary Information [file 41467_2024_55714_MOESM1_ESM.pdf]

Restoring small water bodies to improve lake and river water quality in China

Wangzheng Shen[1,2,3], Liang Zhang[1,3]*, Emily A. Ury[4,5], Sisi Li[1,3], Biqing Xia[1,3], Nandita B. Basu[2,4,6]*

[1]Key Laboratory for Environment and Disaster Monitoring and Evaluation of Hubei, Jianghan Plain-Honghu Lake Station for Wetland Ecosystem Research, Innovation Academy for Precision Measurement Science and Technology, Chinese Academy of Sciences, Wuhan 430077, China

[2]Department of Civil and Environmental Engineering, University of Waterloo, Waterloo, Ontario N2L 3G1, Canada

[3]University of Chinese Academy of Sciences, Beijing 100049, China

[4]Department of Earth and Environmental Sciences, University of Waterloo, Waterloo, Ontario N2L 3G1, Canada

[5]Environmental Defense Fund, New York, NY 10010, USA

[6]Water Institute, University of Waterloo, Waterloo, Ontario N2L 3G1, Canada

*Corresponding author: Liang Zhang (lzhang@apm.ac.cn), Nandita B. Basu (nandita.basu@uwaterloo.ca)

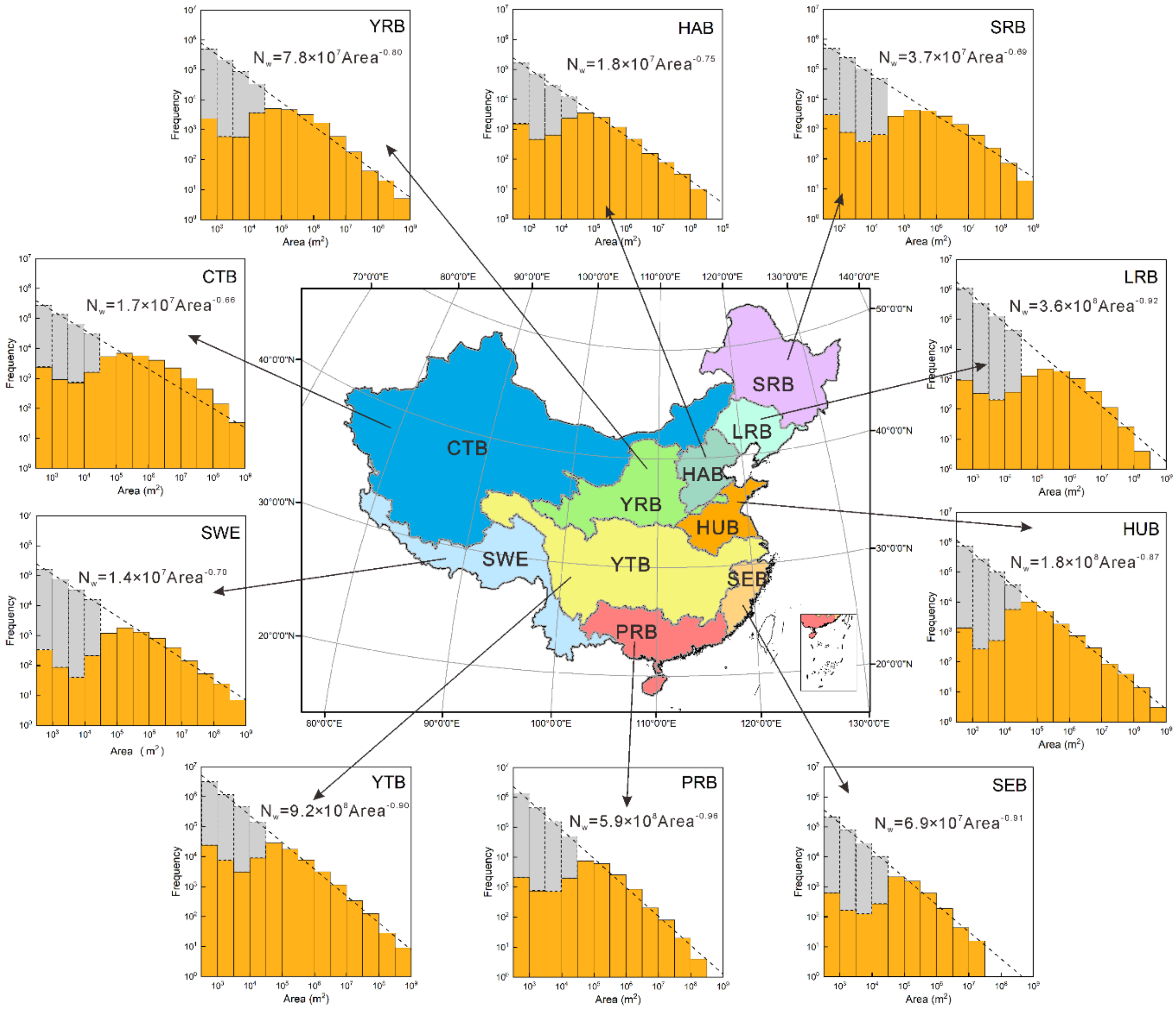

Supplementary Fig. 1 Size frequency distribution of all water bodies across the ten first-order river basins in China. The orange bars in the plots show the water body size distribution in 2015. The dashed lines are the results of fitting a power function. In SRB, LRB, SWE, and CTB, the power function was fitted by water body area greater than 10^5 m^2, and other watersheds were fitted by water body greater than $10^{4.5}$ m^2. The gray bars represent the expected distribution of small water bodies and their potential loss prior to 2015. The 'N_w' in the equation represents the number of water bodies. The name for the first-order watersheds: Songhua River Basin (SRB), Liaohe River Basin (LRB), Haihe River Basin (HAB), Yellow River Basin (YRB), Huaihe River Basin (HUB), Yangtze River Basin (YTB), Southeast Basin (SEB), Pearl River Basin (PRB), Southwest Basin (SWE), Continental Basin (CTB).

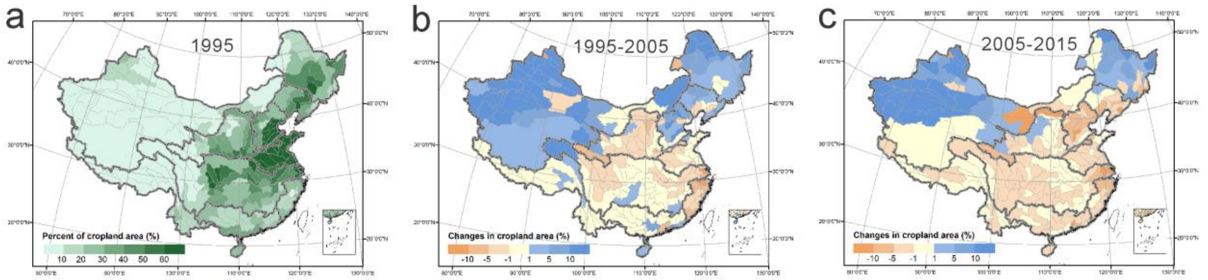

Supplementary Fig. 2 Cropland expansion in China. a, Cropland area density (%) in 1995. b, Percent change in cropland density (%) from 1995 to 2005. c, Percent change in cropland density (%) from 2005 to 2015.

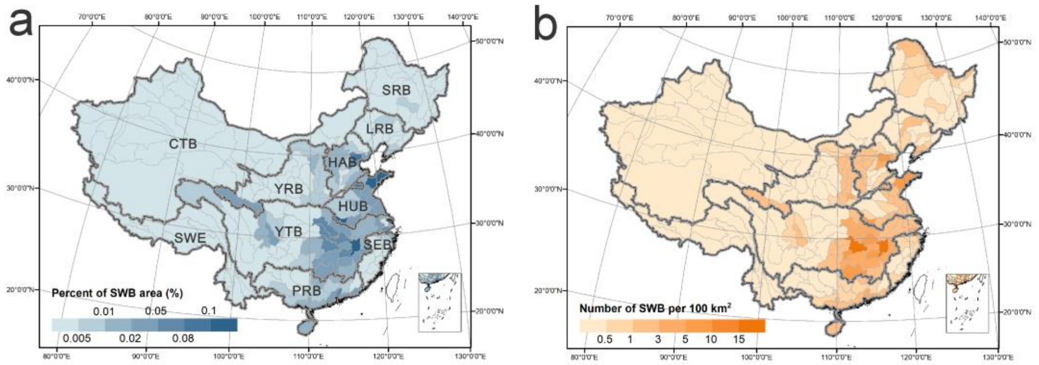

Supplementary Fig. 3 Small water body (SWB) density across China in 2015. a, Small water body extent (percent area) within each of China's third-order river basins. b, Number of small water bodies per 100 km^2. The thick gray lines demarcate the boundaries for the first-order watersheds: Songhua River Basin (SRB), Liaohe River Basin (LRB), Haihe River Basin (HAB), Yellow River Basin (YRB), Huaihe River Basin (HUB), Yangtze River Basin (YTB), Southeast Basin (SEB), Pearl River Basin (PRB), Southwest Basin (SWE), Continental Basin (CTB).

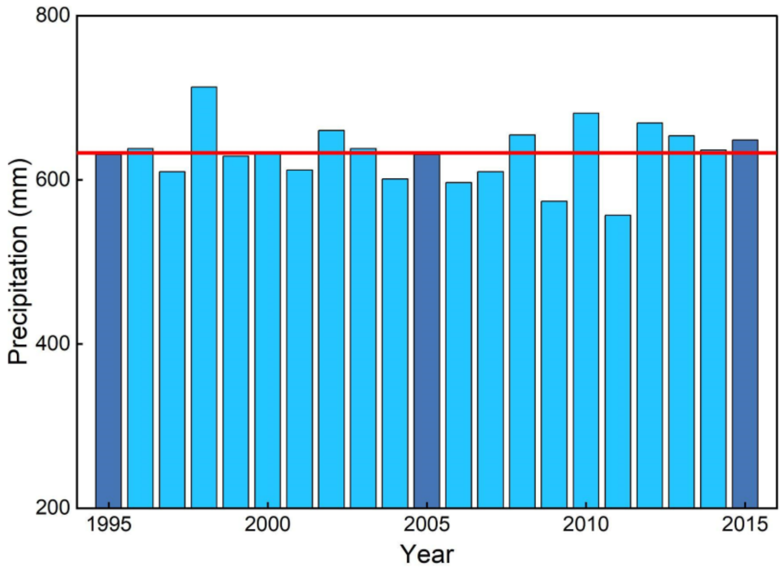

Supplementary Fig. 4. Annual precipitation (mm) across China. The red line represents the multi-year average annual precipitation. The three years of this study (1995, 2005, 2015) are indicated in dark blue. The 1995, 2005, and 2015 precipitation in China is very close to the multi-year average precipitation.

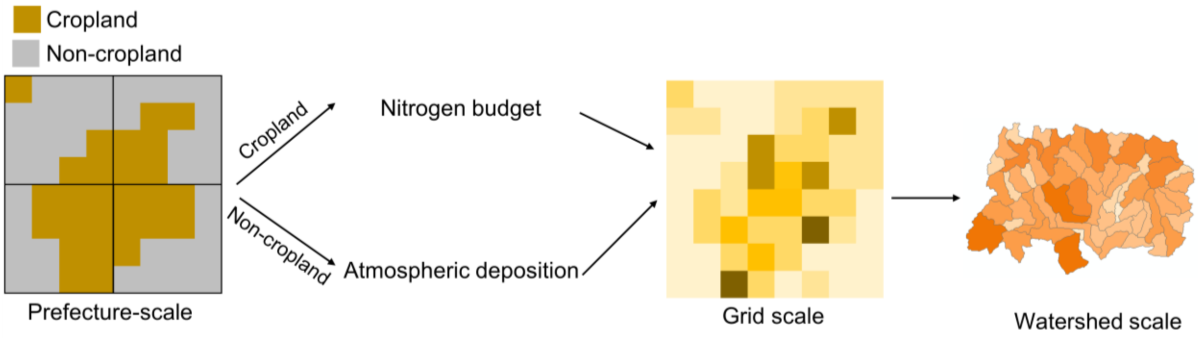

Supplementary Fig. 5. The scale transformation process of nitrogen surplus. The grid scale for the transformation process is 300-m, and the data is sourced from the Multi-Period Land Use and Land Cover Remote Sensing Monitoring Data Set (CNLUCC).

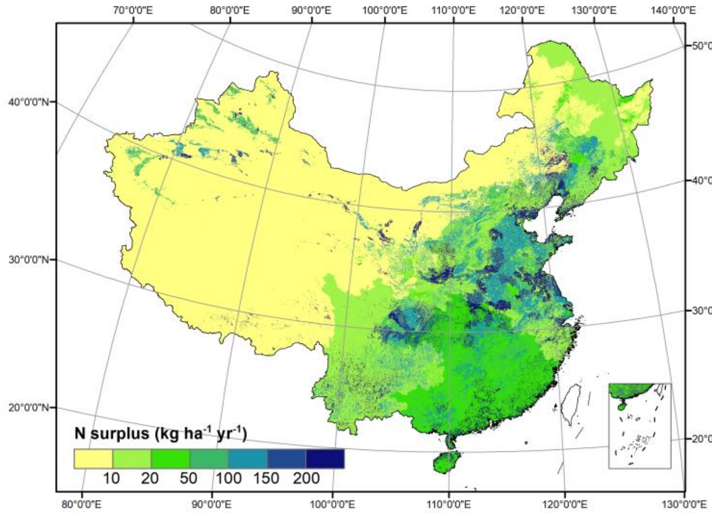

Supplementary Fig. 6. Grid-scale nitrogen surplus data across China in 2015.

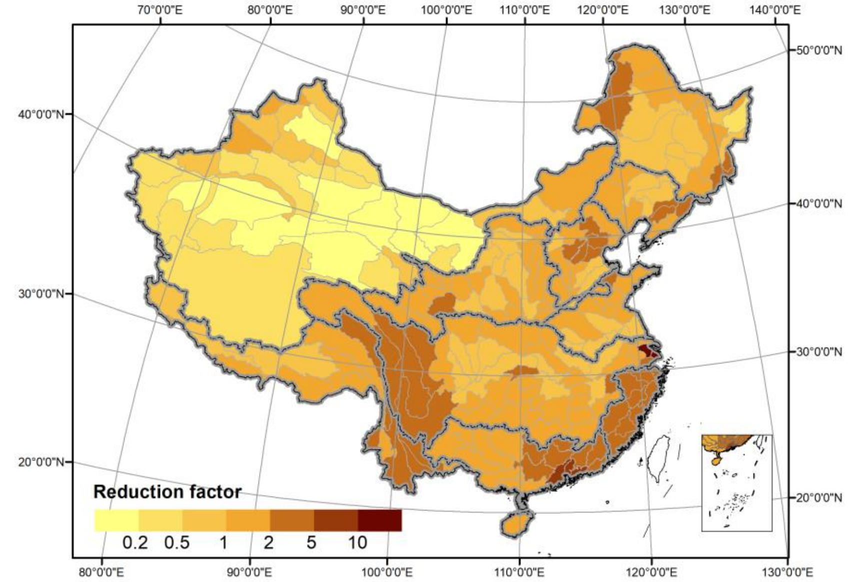

Supplementary Fig. 7. Reduction factor at different watersheds.

Supplementary Table 1 The annual change in the number and area of small water bodies

Basin	The average change in the number of small water bodies (yr^{-1})	The average change in the area of small water bodies ($km^2\ yr^{-1}$)
Songhua River Basin	-1041	-2.3
Liaohe River Basin	-159	-0.2
Haihe River Basin	-59	0.8
Yellow River Basin	-1783	-3.9
Huaihe River Basin	-69	1.3
Yangtze River Basin	-3680	-12.6
Southeast Basin	-4	-0.3
Pearl River Basin	-218	-0.2
Southwest Basin	-371	-1.4
Continental Basin	-2612	-5.9

Note: The average result refers to the average from 1995 to 2015.

Supplementary Table 2 Basin area and the percentage of different land use types

Basin	Area (km^2)	The percentage of land use (%)			
		Paddy field	Upland	Forest and grassland	Constructed land
Songhua River Basin	9.5×10^6	5.7	23.7	59.2	2.1
Liaohe River Basin	3.2×10^6	3.1	32.7	50.6	5.3
Haihe River Basin	3.2×10^6	0.4	46.8	38.1	11.4
Yellow River Basin	8.0×10^6	0.8	24.5	62.1	3.2
Huaihe River Basin	3.3×10^6	15.1	52.4	11.0	16.1
Yangtze River Basin	1.8×10^7	13.2	13.6	64.6	2.7
Southeast Basin	2.0×10^6	14.4	4.5	74.3	5.0
Pearl River Basin	5.8×10^6	11.2	11.4	71.3	3.7
Southwest Basin	9.2×10^6	0.8	4.0	74.3	0.2
Continental Basin	3.7×10^7	0.003	4.0	37.0	0.4

Note: The land use data is based on the results from 2015.

Supplementary Table 3 The nitrogen loss rate for different land use types

Land use type		Nitrogen loss rate (%)
Paddy field	Synthetic fertilizer	5.2
	Livestock manure	5.0
Dryland	Synthetic fertilizer	3.5 (North), 11.0 (South)
	Livestock manure	5.0
Forest/grassland		22.5
Constructed land		75

Note: South includes Yangtze River Basin, Pearl River Basin, Southeast Basin, and Southwest Basin; North includes Continental Basin, Songhua River Basin, Liaohe River Basin, Haihe River Basin, Huaihe River Basin, and Yellow River Basin.

Supplementary Table 4 The total area of water bodies restored in the different basins for the scenario analysis. The area restored is estimated as the grey area in the size-frequency distribution (Extended Figure 1), which corresponds to our estimate for the smaller water bodies lost in each basin

Basin	Area (km^2)	Increased in total areal coverage of water bodies (%)	Number of restored small water bodies of different sizes (Scenario 1)			
			$10^{2.5}$-10^3 m^2	10^3-$10^{3.5}$ m^2	$10^{3.5}$-10^4 m^2	10^4-$10^{4.5}$ m^2
Songhua River Basin	2000	3	479033	218007	98932	44433
Liaohe River Basin	2680	28	1039021	359425	124250	42685
Haihe River Basin	531	7	157274	66901	27937	9809
Yellow River Basin	1605	8	506367	203119	80992	29065
Huaihe River Basin	1932	13	718856	264565	96866	30230
Yangtze River Basin	8231	17	3109987	1108245	394239	132447
Southeast Basin	589	30	222727	78630	27673	9535
Pearl River Basin	3120	36	1319427	435338	143184	45532
Southwest Basin	684	4	169877	75855	33843	14906
Continental Basin	1191	1	272106	128215	60031	27019
